# Supplementary material for: GlRac regulates an ATG8-independent autophagy-like pathway in Giardia lamblia
Source: PLoS Pathog. 2026 Oct 6;22(10):e1014624. doi: 10.1371/journal.ppat.1014624 (PMC13657398; doi:10.1371/journal.ppat.1014624)
Supplement: Supplementary file 1 [file ppat.1014624.s019.docx]

**Striking Image Captions**

**Striking Image 1**

**Caption:** An autophagosome-like compartment in *Giardia lamblia*. Halo-*Gl*Rac (magenta) marks the compartment, which is labeled by the active cysteine protease probe BODIPY-LHVS (cyan). Dextran Texas Red (yellow) marks peripheral vacuoles.

**Image credit:** Paredez Lab, University of Washington.

**Striking Image 2**

**Caption:** Autophagosome-like compartments in *Giardia lamblia*. Halo-*Gl*Rac marks a circular autophagosome-like compartment (top), while transmission electron microscopy reveals the double-membrane organization of these compartments (bottom).

**Image credit:** Paredez Lab, University of Washington.
