# Supplementary material for: GlRac regulates an ATG8-independent autophagy-like pathway in Giardia lamblia
Source: PLoS Pathog. 2026 Oct 6;22(10):e1014624. doi: 10.1371/journal.ppat.1014624 (PMC13657398; doi:10.1371/journal.ppat.1014624)
Supplement: Peer review history

# Peer review: Decision Letter 0

**DOI:** 10.1371/journal.ppat.1014624.r001

**Authors:** Dominique Soldati-Favre, Carmen Faso

26 May 2026

PPATHOGENS-D-26-00644

Gl Rac regulates a noncanonical, ATG8-independent autophagy-like pathway in Giardia lamblia

PLOS Pathogens

Dear Dr. Paredez,

Thank you for submitting your manuscript to PLOS Pathogens. After careful consideration, we feel that it has merit but does not fully meet PLOS Pathogens's publication criteria as it currently stands. Therefore, we invite you to submit a revised version of the manuscript that addresses the points raised during the review process.

Please submit your revised manuscript by Jul 25 2026 11:59PM. If you will need more time than this to complete your revisions, please reply to this message or contact the journal office at plospathogens@plos.org. When you're ready to submit your revision, log on to https://www.editorialmanager.com/ppathogens/ and select the 'Submissions Needing Revision' folder to locate your manuscript file.

Please include the following items when submitting your revised manuscript:

* A letter that responds to each point raised by the editor and reviewer(s). You should upload this letter as a separate file labeled 'Response to Reviewers'. This file does not need to include responses to any formatting updates and technical items listed in the 'Journal Requirements' section below.

* A marked-up copy of your manuscript that highlights changes made to the original version. You should upload this as a separate file labeled 'Revised Manuscript with Track Changes'.

* An unmarked version of your revised paper without tracked changes. You should upload this as a separate file labeled 'Manuscript'.

If you would like to make changes to your financial disclosure, competing interests statement, or data availability statement, please make these updates within the submission form at the time of resubmission. Guidelines for resubmitting your figure files are available below the reviewer comments at the end of this letter. As the corresponding author, your ORCID iD is verified in the submission system and will appear in the published article. PLOS supports the use of ORCID, and we encourage all coauthors to register for an ORCID iD and use it as well. Please encourage your coauthors to verify their ORCID iD within the submission system before final acceptance, as unverified ORCID iDs will not appear in the published article. Only the individual author can complete the verification step; PLOS staff cannot verify ORCID iDs on behalf of authors.

We look forward to receiving your revised manuscript.

Kind regards,

Carmen Faso, Ph.D.

Academic Editor

PLOS Pathogens

Dominique Soldati-Favre

Section Editor

PLOS PathogensSumita Bhaduri-McIntosh

Editor-in-Chief

PLOS Pathogens

orcid.org/0000-0003-2946-9497

Michael Malim

Editor-in-Chief

PLOS Pathogens

orcid.org/0000-0002-7699-2064

**Additional Editor Comments**

Dear Dr Paredez,

Two reviewers have both carefully evaluated your manuscript and both request further analysis, leading to a decision for major revision before consideration for acceptance.

These are summarized as follows:

-Provide decisive functional evidence that GlRac-positive compartments represent an autophagy-like pathway with degradative activity.

-Resolve distinction and relationship between GlRac compartments and peripheral vacuoles.

-Include pathway inhibition experiments to validate molecular roles and organelle identity.

-Improve figures and add schematic models for clarity.

-Ensure consistent nomenclature and precise terminology.

-Add rigorous quantitative or bioinformatic analyses to support molecular claims.

Thank you for addressing these requests,

Carmen Faso

**Journal Requirements:**

If the reviewer comments include a recommendation to cite specific previously published works, please review and evaluate these publications to determine whether they are relevant and should be cited. There is no requirement to cite these works unless the editor has indicated otherwise.

1) We ask that a manuscript source file is provided at Revision. Please upload your manuscript file as a .doc, .docx, .rtf or .tex. If you are providing a .tex file, please upload it under the item type u2018LaTeX Source Fileu2019 and leave your .pdf version as the item type u2018Manuscriptu2019.

2) We noticed that you used the phrase 'data not shown' in "page no 12" line number 318 in the manuscript. We do not allow these references, as the PLOS data access policy requires that all data be either published with the manuscript or made available in a publicly accessible database. Please amend the supplementary material to include the referenced data or remove the references.

3) We do not publish any copyright or trademark symbols that usually accompany proprietary names, eg ©,  ®, or TM  (e.g. next to drug or reagent names). Therefore please remove all instances of trademark/copyright symbols throughout the text, including:

- ® on Line number 705, page no 27

- TM on page no 26, line no 690 and 693 and page no 52.

4) Please upload all main figures as separate Figure files in .tif or .eps format. For more information about how to convert and format your figure files please see our guidelines:

https://journals.plos.org/plospathogens/s/figures

5) Please upload a copy of [Figures 1-6] which you refer to in your text on pages [5, 7, 9, 13, 14, and 17]. Or, if the figure is no longer to be included as part of the submission please remove all reference to it within the text.

6) We notice that your supplementary Figures [S1-S8 Figures] are included in the manuscript file. Please remove them and upload them with the file type 'Supporting Information'. Please ensure that each Supporting Information file has a legend listed in the manuscript after the references list.

7) Please amend your detailed Financial Disclosure statement. This is published with the article. It must therefore be completed in full sentences and contain the exact wording you wish to be published.

1) State what role the funders took in the study. If the funders had no role in your study, please state: "The funders had no role in study design, data collection and analysis, decision to publish, or preparation of the manuscript."

2) If any authors received a salary from any of your funders, please state which authors and which funders..

If you did not receive any funding for this study, please simply state: u201cThe authors received no specific funding for this work.u201d

8. Thank you for submitting your data to a repository. We have been unable to access the data using the DOI link "https://doi.org/10.6084/m9.figshare.31366717" you provided. Please contact the repository to ensure that the accession numbers are valid. /. If a link  needs updating, please provide a revised Data Availability Statement including the information.

9. PLOS now requires that authors provide the original uncropped and unadjusted images underlying all blot or gel results reported in a submission’s figures or Supporting Information files. This policy and the journal’s other requirements for blot/gel reporting and figure preparation are described in detail at https://journals.plos.org/plosone/s/figures#loc-blot-and-gel-reporting-requirements and https://journals.plos.org/plosone/s/figures#loc-preparing-figures-from-image-files.

When you submit your revised manuscript, please ensure that your figures adhere fully to these guidelines and provide the original underlying images for all blot or gel data reported in your submission. See the following link for instructions on providing the original image data: https://journals.plos.org/plosone/s/figures#loc-original-images-for-blots-and-gels.

In your cover letter, please note whether your blot/gel image data are in Supporting Information or posted at a public data repository, provide the repository URL if relevant, and provide specific details as to which raw blot/gel images, if any, are not available. Email us at customercare@plos.org if you have any questions.

10. We have amended your Competing Interest statement to comply with journal style. We kindly ask that you double check the statement and let us know if anything is incorrect.

**Reviewers' Comments:**

Reviewer's Responses to Questions

**Part I - Summary**

Please use this section to discuss strengths/weaknesses of study, novelty/significance, general execution and scholarship.

Reviewer #1: The manuscript by Klippel et al. addresses a fundamental question in evolutionary cell biology: how the highly divergent parasite Giardia lamblia, which lacks most canonical ATG genes, performs intracellular recycling.

Strengths and Significance: The significance of this study lies in the precise presentation of morphological data, including high-resolution transmission electron microscopy (TEM) and live-cell imaging of stress-responsive organelle formation in Giardia. The visual documentation of these compartments forming de novo provides strong evidence for the existence of a regulated membrane trafficking event. Furthermore, the finding that these organelles are directly regulated by GlRac—the sole Rho GTPase in Giardia—is highly novel. This provides a key molecular handle for understanding an unconventional intracellular degradation system that does not depend on the canonical ATG8.

Weaknesses and Scholarship: However, the primary weakness is the logical leap in the Results section, where the authors refer to these compartments as "autophagy-like" without direct evidence of cargo degradation. Specifically, there is insufficient evidence to show whether the GlRac-positive structures are directly involved in intracellular degradation or if they exhibit behaviors distinctly different from existing peripheral vacuoles (PVs). If these structures cannot be distinguished from existing PVs, this study should be described as the discovery of a new function for PVs rather than the discovery of autophagy. It may also be important for the authors to present a schematic diagram of their proposed model. Until decisive evidence is provided showing that GlRac-positive structures behave differently from canonical PVs, the use of the term "autophagy-like" must be avoided.

Reviewer #2: The article entitled “GlRac regulates a noncanonical, ATG8-independent autophagy-like pathway in Giardia lamblia” provides a comprehensive study describing a functional, ATG8-independent autophagic process in the protozoan parasite Giardia lamblia.

The authors identify that the sole Rho family protein in this organism, GlRac, regulates the formation and degradation of autophagic-like structures. These GlRac-positive structures increase during encystation and nutrient deprivation. Interestingly, they are disrupted by the anti-giardial drug quinacrine, mimicking the inhibition of autophagic flux observed in mammalian cells treated with this compound.

Using molecular, biochemical, and cell biology approaches, the authors demonstrate the lack of function of the putative ATG8 protein. Simultaneously, they provide evidence for actin remodeling, acidification of autophagic-like compartments (derived from peripheral vesicles), and degradative activity.

The primary weaknesses relate to the inconclusive data regarding the distribution of ATG proteins under nutrient deprivation (specifically the partial colocalization with autophagic-like structures) and the lack of functional studies confirming the involvement of the GlTOR pathway.

Overall, this is a novel and significant work that requires a few targeted experiments to strengthen the conclusions for publication.

**********

**Part II – Major Issues: Key Experiments Required for Acceptance**

Please use this section to detail the key new experiments or modifications of existing experiments that should be absolutely required to validate study conclusions.

Generally, there should be no more than 3 such required experiments or major modifications for a "Major Revision" recommendation. If more than 3 experiments are necessary to validate the study conclusions, then you are encouraged to recommend "Reject".

Reviewer #1: This manuscript lacks critical data to conclude an "autophagy-like" process. Unless the following points are clearly addressed, the term "autophagy-like" should not be used in the title or the Results section.

1. Decisive distinction from Peripheral Vacuoles (PVs) and evidence of intracellular degradation

In Giardia, PVs are well-established as acidic and degradative organelles. The authors must clearly describe and provide data showing the differences between GlRac-positive structures and existing PVs. Specifically, the Introduction should also mention the PV system. Furthermore, it is essential to provide decisive evidence that these structures exhibit behaviors distinct from canonical PVs under various culture conditions and drug treatments. Alternatively, providing data that demonstrates GlRac-dependent intracellular degradation would significantly strengthen the authors' claims (though the reviewer understands the technical difficulties of directly proving proteolysis in parasitic protozoa).

Required action: Provide evidence showing the spatial and functional relationship between GlRac and established PVs. If these structures cannot be distinguished from PVs, the study should be reframed as the discovery of a new function for the PV system.

2. Rigorous bioinformatic and phylogenetic analysis of candidate autophagy factors

The manuscript identifies and localizes several key autophagy-related candidates: GlATG1.1, GlATG1.2, GlVPS15, GlVPS34, GlATG18, GlATG21, GlATG16, ATG7, ATG8, and GTOR. Given the divergent nature of Giardia proteins, casual identification of these factors may negatively impact future research in the field.

Required action: For all factors listed above, the authors must perform amino acid sequence alignments and phylogenetic analyses. To discuss this work in the context of autophagy, it is crucial to provide objective evidence and evolutionary validation for identifying these proteins as homologs. If the phylogenetic evidence is insufficient, the use of these names should be reconsidered or qualified with more conservative terminology.

3. Presentation of a schematic model diagram

A visual summary is necessary to clarify the findings and the proposed non-canonical autophagy pathway.

Required action: Provide a schematic diagram illustrating the entire process, including GlRac-mediated regulation, membrane formation, the relationship with PVs, and the presumed degradation steps. This will help readers better understand the novelty and the core claims of the study.

Reviewer #2: 1. PI3K Inhibition: Given that the role of GlTOR signaling remains inconclusive, and considering the presence of Vps34 and Vps15 homologs in Giardia, the authors should investigate the effect of PI3K inhibitors (such as wortmannin or 3-methyladenine) on the size, quantity, and presence of GlRac structures during nutrient deprivation.

2. Is the GlRac pathway sensitive to Bafilomycin A1? Testing if a V-ATPase inhibitor (BafA1) blocks the degradation within these GlRac structures would definitively prove that these compartments are lysosome-like organelles.

3. Cargo Identification: To confirm the functional role of this pathway, it is essential to identify a potential cargo. Demonstrating the degradation of a specific target molecule or cellular component via this autophagic-like process—particularly during encystation—would be a key experiment to validate the study’s functional claims.

________________________________________

**********

**Part III – Minor Issues: Editorial and Data Presentation Modifications**

Please use this section for editorial suggestions as well as relatively minor modifications of existing data that would enhance clarity.

Reviewer #1: 1. Expansion of the Introduction regarding PVs and proteases: As noted in Part I, the Introduction should include a more comprehensive review of Giardia’s existing endolysosomal system. Specifically, citing seminal works on PV acidification and the roles of cysteine proteases (cathepsins) in the Giardia life cycle is necessary to properly contextualize the discovery of GlRac-positive vesicles.

2. Rationale for focusing on GlRac: The authors should explicitly state in the Introduction why GlRac was selected as a candidate regulator. Mentioning its unique status as the sole Rho GTPase in Giardia and its known involvement in cytoskeletal dynamics would help bridge the logical gap between the general background and the first Results section.

3. Refinement of the term "Autophagosome-like": Throughout the Results section, the authors frequently use the term "autophagosome-like." To maintain scientific objectivity, the reviewer suggests using more descriptive and neutral terms, such as "GlRac-positive compartments" or "GlRac-positive vesicles," until the functional evidence requested in the Major Issues is fully provided.

4. Figure 5E: Please replace the numbers with the corresponding protein names to improve clarity for the reader.

5. Line 294: Please provide a brief explanation or definition for "RMSD."

6. Line 515: Since Toxoplasma is a monotypic genus, "spp." should be corrected to "sp."

Reviewer #2: • Colocalization Analysis: The colocalization studies with ATG proteins (Fig. S4) are not entirely convincing. Have the authors performed quantitative analysis using Pearson’s or Manders’ colocalization coefficients?

• Figure Annotations: Peripheral vesicles (PVs) should be clearly labeled/indicated in Fig. S2.

• Nomenclature Consistency: The authors alternate between the terms “older culture media (48, 72, or 96 h)” and “starvation conditions.” Please clarify if these refer to the same experimental state and unify the nomenclature throughout the manuscript.

• Typos: In Line 456, the word “Fig.” is repeated.

**********

PLOS authors have the option to publish the peer review history of their article (what does this mean?). If published, this will include your full peer review and any attached files.

If you choose “no”, your identity will remain anonymous but your review may still be made public.

**Do you want your identity to be public for this peer review?**  For information about this choice, including consent withdrawal, please see our Privacy Policy.

Reviewer #1: No

Reviewer #2: No

[NOTE: If reviewer comments were submitted as an attachment file, they will be attached to this email and accessible via the submission site. Please log into your account, locate the manuscript record, and check for the action link "View Attachments". If this link does not appear, there are no attachment files.]

**Figure resubmission:**

While revising your submission, we strongly recommend that you use PLOS’s NAAS tool (https://ngplosjournals.pagemajik.ai/artanalysis) to test your figure files. NAAS can convert your figure files to the TIFF file type and meet basic requirements (such as print size, resolution), or provide you with a report on issues that do not meet our requirements and that NAAS cannot fix.

After uploading your figures to PLOS’s NAAS tool - https://ngplosjournals.pagemajik.ai/artanalysis, NAAS will process the files provided and display the results in the "Uploaded Files" section of the page as the processing is complete. If the uploaded figures meet our requirements (or NAAS is able to fix the files to meet our requirements), the figure will be marked as "fixed" above. If NAAS is unable to fix the files, a red "failed" label will appear above. When NAAS has confirmed that the figure files meet our requirements, please download the file via the download option, and include these NAAS processed figure files when submitting your revised manuscript.

**Reproducibility:**

To enhance the reproducibility of your results, we recommend that authors of applicable studies deposit laboratory protocols in protocols.io, where a protocol can be assigned its own identifier (DOI) such that it can be cited independently in the future. Additionally, PLOS ONE offers an option to publish peer-reviewed clinical study protocols. Read more information on sharing protocols at https://plos.org/protocols?utm_medium=editorial-email&utm_source=authorletters&utm_campaign=protocols

# Author comment: Author response to Decision Letter 1

**DOI:** 10.1371/journal.ppat.1014624.r002

24 Jul 2026

Attachment Submitted filename: PLOS_Striking_Image_Captions.docx

# Peer review: Decision Letter 1

**DOI:** 10.1371/journal.ppat.1014624.r003

**Authors:** Dominique Soldati-Favre, Carmen Faso

31 Aug 2026

Dear Dr. Paredez,

I am pleased to inform you and all co-authors that your manuscript, "Gl Rac regulates an ATG8-independent autophagy-like pathway in Giardia lamblia", has been editorially accepted for publication at PLOS Pathogens.

Both reviewers, as you can also read below, find your revised manuscript significantly improved.

Before your manuscript can be formally accepted and sent to production, you will need to complete our formatting changes, which you will receive by email within a week. Please note that your manuscript will not be scheduled for publication until you have made the required changes.

IMPORTANT NOTES

(1) Copyediting and Proofreading: The corresponding author will receive a typeset proof for review, to ensure errors have not been introduced during production. Please review the PDF proof of your manuscript carefully, as this is the last chance to correct any errors. Please note that major changes, or those which affect the scientific understanding of the work, will likely cause delays to the publication date of your manuscript.

(2) Appropriate Figure Files: Please remove all name and figure # text from your figure files. Please also take this time to check that your figures are of high resolution, which will improve the readbility of your figures and help expedite your manuscript's publication. Please note that figures must have been originally created at 300dpi or higher. Do not manually increase the resolution of your files. For instructions on how to properly obtain high quality images, please review our Figure Guidelines, with examples at: http://journals.plos.org/plospathogens/s/figures.

We encourage you to use PACE (http://pace.apexcovantage.com/), a figure-checking tool which helps authors ensure all figures meet PLOS requirements so that figures can publish in the highest quality. PACE is a digital diagnostic and conversion tool for figure files. It will provide information about any failed check(s) and, if able, will automatically convert the figure into an acceptable file that passes quality checks.

To use PACE, you must first register as a user. Then, login and navigate to the UPLOAD tab, where you will find detailed instructions on how to use the tool.

(3) Striking Image: Please upload a striking still image to accompany your article if one is available (you can include a new image or an existing one from within your manuscript). Should your paper be accepted, this image will be considered for our monthly issue image and may also appear on our website to feature your article. Please upload this as a separate file, selecting "striking image" as the file type upon upload. Please also include a separate "Other" file with a caption, including credits and any potential copyright information. Please do not include the caption in the main article file. If your image is from someone other than yourself, please ensure that the artist has read and agreed to the terms and conditions of the Creative Commons Attribution License at http://journals.plos.org/plospathogens/s/content-license. Please note that PLOS cannot publish copyrighted images.

(4) Press Release or Related Media: If you or your institution will be preparing press materials for this manuscript, please inform our press team in advance at plospathogens@plos.org as soon as possible. We ask that you contact us within one week to plan ahead of our fast Production schedule. If you need to know your paper's publication date for related media purposes, you must coordinate with our press team, and your manuscript will remain under a strict press embargo until the publication date and time.

(5) Update your Profile Information: Now that your manuscript has been provisionally accepted, please log into Editorial Manager and update your profile, if needed. Go to http://www.editorialmanager.com/ppathogens, log in, and click on the "Update My Information" link at the top of the page. Please update your user information to ensure an efficient production and billing process.

(6) LaTeX users only: Our staff will ask you to upload a TEX file in addition to the PDF before the paper can be sent to typesetting, so please carefully review our Latex Guidelines http://journals.plos.org/plospathogens/s/latex in the meantime.

Thank you again for supporting Open Access publishing; we are looking forward to publishing your work in PLOS Pathogens.

Best regards,

Carmen Faso, Ph.D.

Academic Editor

PLOS Pathogens

Dominique Soldati-Favre

Editor-in-Chief

PLOS Pathogens

***********************************************************

Reviewer's Responses to Questions

**Part I - Summary**

Please use this section to discuss strengths/weaknesses of study, novelty/significance, general execution and scholarship.<br/><br/>

Reviewer #1: In this revised manuscript, Klippel et al. have exceptionally addressed all of my concerns regarding the functional validation and characterization of the GlRac-positive compartments in Giardia lamblia.

The authors provided compelling new evidence demonstrating active degradation function, cleared up the spatial and functional relationships between these compartments and the peripheral vacuoles (PVs), and confirmed their predominantly ER-derived nature. Crucially, the authors responsibly revised their bioinformatic annotations by performing a rigorous reciprocal BLAST re-analysis and withdrawing claims for unsupported ATG candidates. The newly added summary model also effectively clarifies the pathway while distinguishing established steps from open questions. Overall, the authors have successfully validated their conclusions and significantly strengthened the manuscript.

Reviewer #2: The revised version of the manuscript 'GlRac regulates a noncanonical, ATG8-independent autophagy-like pathway in Giardia lamblia' is significantly improved. The authors have conducted new experiments that address nearly all of the original concerns, particularly those regarding the acidic and hydrolytic activity of the GlRac-positive compartments using the BODIPY-LHVS probe and concanamycin treatment. Additionally, the authors provided a clear explanation for the colocalization studies and their rationale for excluding PI3K inhibition experiments. In summary, I recommend accepting this manuscript in its current form.

--------------------

**Part II – Major Issues: Key Experiments Required for Acceptance**

Please use this section to detail the key new experiments or modifications of existing experiments that should be absolutely required to validate study conclusions.<br/><br/>Generally, there should be no more than 3 such required experiments or major modifications for a "Major Revision" recommendation. If more than 3 experiments are necessary to validate the study conclusions, then you are encouraged to recommend "Reject".<br/><br/>

Reviewer #1: None.

The authors have thoroughly addressed all of my major requests with high-quality experimental data and revised analyses. No further experiments are required.

Reviewer #2: (No Response)

--------------------

**Part III – Minor Issues: Editorial and Data Presentation Modifications**

Please use this section for editorial suggestions as well as relatively minor modifications of existing data that would enhance clarity.<br/><br/>

Reviewer #1: None.

Reviewer #2: (No Response)

--------------------

PLOS authors have the option to publish the peer review history of their article (what does this mean?). If published, this will include your full peer review and any attached files.

If you choose “no”, your identity will remain anonymous but your review may still be made public.

**Do you want your identity to be public for this peer review?**  For information about this choice, including consent withdrawal, please see our Privacy Policy.

Reviewer #1: Yes: Hirokazu Sakamoto

Reviewer #2: Yes: Patricia Silvia Romano

# Editor report: Acceptance letter

**DOI:** 10.1371/journal.ppat.1014624.r004

**Authors:** Dominique Soldati-Favre, Carmen Faso

Dear Dr. Paredez,

We are delighted to inform you that your manuscript, "GlRac regulates an ATG8-independent autophagy-like pathway in Giardia lamblia

," has been formally accepted for publication in PLOS Pathogens.

We have now passed your article onto the PLOS Production Department who will complete the rest of the pre-publication process. All authors will receive a confirmation email upon publication.

Thank you again for supporting open-access publishing; we are looking forward to publishing your work in PLOS Pathogens.

Best regards,

Dominique Soldati-Favre

Editor-in-Chief

PLOS Pathogens
